# Rab11-FIP1/RCP Functions as a Major Signalling Hub in the Oncogenic Roles of Mutant p53 in Cancer

**DOI:** 10.3389/fonc.2021.804107

**Published:** 2021-12-21

**Authors:** Yannick von Grabowiecki, Vinaya Phatak, Lydia Aschauer, Patricia A. J. Muller

**Affiliations:** ^1^ Tumour Suppressors Group, Cancer Research United Kingdom (UK) Manchester Institute, The University of Manchester, Macclesfield, United Kingdom; ^2^ Medical Research Council (MRC) Toxicology Unit, Cambridge, United Kingdom; ^3^ Avacta Life Sciences, Cambridge, United Kingdom; ^4^ Orbit Discovery, Oxford, United Kingdom; ^5^ Department of Biosciences, Faculty of Science, Durham University, Durham, United Kingdom

**Keywords:** Rab11-FIP1, RCP, p53, recycling, cancer, integrin, invasion, metastasis

## Abstract

Rab11-FIP1 is a Rab effector protein that is involved in endosomal recycling and trafficking of various molecules throughout the endocytic compartments of the cell. The consequence of this can be increased secretion or increased membrane expression of those molecules. In general, expression of Rab11-FIP1 coincides with more tumourigenic and metastatic cell behaviour. Rab11-FIP1 can work in concert with oncogenes such as mutant p53, but has also been speculated to be an oncogene in its own right. In this perspective, we will discuss and speculate upon our observations that mutant p53 promotes Rab11-FIP1 function to not only promote invasive behaviour, but also chemoresistance by regulating a multitude of different proteins.

## Introduction

**Figure 1:**
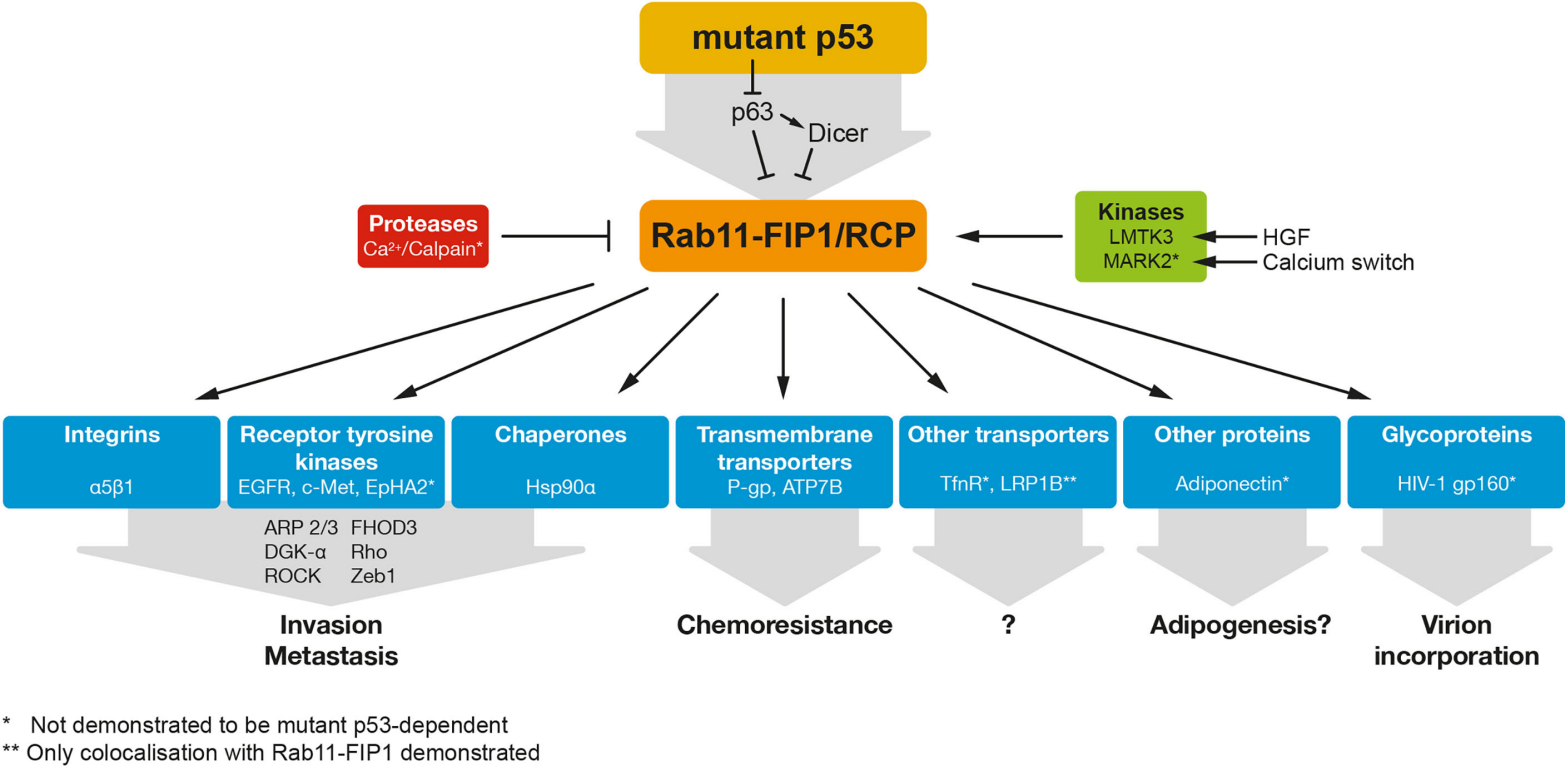
Mutant p53 regulates RAB11-FIP1-dependent re-localisation of a variety of proteins. Mutant p53 can regulate RAB11-FIP1 by inhibiting the p53 family member p63 and/or the downstream target Dicer. Rab11-FIP1 enhances the re-localisation of a variety of proteins indicated in this figure. * indicates mutant p53 dependency. ** indicates only co-localisation demonstrated with Rab11-FIP1.

Rab11-FIP1 was identified as a downstream effector and interactor of the Rab-GTPase Rab11a, important in membrane recycling systems (1). Rab GTPases form a family of more than 70 members, regulating vesicle trafficking in different cell localisations or compartments (2–4), and cycle between a membrane-bound state (bound to GTP) and a cytosolic state (free of GTP). Rab11 specifically, has been shown active and involved in endocytosis, recycling compartments and the trans-golgi network, regulating endocytic membrane traffic. When bound to GTP, Rab11 interacts with Rab11-FIP1 in the early endosomal recycling compartment (5). Rab11-FIP1 is required for endosomal recycling, and regulates the sorting of proteins into endosomes and the delivery of cargo to the plasma membrane (6, 7). Its cargo can be diverse and includes receptor tyrosine kinases, integrins and other membrane receptors or molecules schematically depicted in **Figure 1** and discussed below.

Based on the frequent overexpression of Rab11-FIP1 in cancers (overexpression or amplified 8p11–12 amplicon), Rab11-FIP1 was proposed to be an oncogene (8, 9). Importantly, Rab11-FIP1 can drive metastasis *in vivo*, which was demonstrated using a pancreatic ductal adenocarcinoma mouse model harbouring pancreas-specific p53 and K-Ras mutations. In this context, loss of Rab11-FIP1 reduced the overall metastatic burden (10). In contrast, some data suggest that loss of Rab11-FIP1 promotes oncogenesis or invasion in cervical or oesophageal cancers (11, 12). Rab11-FIP1 can therefore not be classed as an oncogene in its own right yet. It is possible that increased Rab11-FIP1 function or expression is context-dependent and enhanced by the presence of oncogenes, including mutant p53, or by a tumour promoting environment in which cytokines, integrins and growth factors such as EGF are enriched (13).

## Mutant p53 and the Role of Rab11-FIP1 in Integrin/RTK Signalling

p53 is a transcription factor involved in many different processes, including cell death and senescence. By reacting to incoming stresses, p53 activates specific transcriptional programmes, such as apoptosis or cell cycle arrest. This allows the organism to stop further accumulation of DNA damage and allows for DNA repair or cell death depending on the amount of stress.

Mutations in *TP53* can lead to loss of p53 protein expression or in about 75% of cases, to the expression of a mutant p53 protein (14). Mutant p53 expression results in loss of the tumour suppressor function as well as acquisition of a gain-of-function that promotes proliferation, invasion, metastasis or chemoresistance. Proposed mechanisms for gain-of-function include binding to new response elements on the DNA and the interaction with many different proteins, including transcription factors such as the p53 family member p63 (15). We have shown previously that mutant p53 inhibits TAp63α function, and in those same conditions, mutant p53 promotes the interaction between integrins, RTKs and the Rab-coupling protein/Rab11-Family Interacting Protein 1 (RCP/Rab11-FIP1), leading to increased invasion, cell scattering, metastasis and chemoresistance (16–18).

Integrins form a family of glycoprotein cell surface receptors that interact with the microenvironment. By binding to extracellular ligands, they promote adhesion to the extracellular matrix, other cells or activate intracellular signalling pathways that are shared and interconnected with receptor tyrosine kinases (RTKs) (19). Integrins are thus involved in a range of cellular processes that promote tumorigenesis and confer a survival advantage to cancer cells. RTKs are cell surface receptors that bind to growth factors, hormones and cytokines to promote cell signalling mediated through their inherent tyrosine kinase activity. The integrin/RTK cooperation amplifies signalling, promoting tumour formation, aggressiveness and drug resistance.

We and others have shown that the cooperation of integrin β1 and EGFR is dependent on Rab11-FIP1 (13, 16, 20, 21). In some cells, this was dependent on stimulation with the EGFR ligand EGF, or with the αvβ3 integrin ligand osteopontin (13). However, when mutant p53 was expressed, osteopontin activation was not required to induce Rab11-FIP1-dependent delivery of integrins and EGFR to the plasma membrane (16). Downstream of EGFR and integrins, the Akt/PKB pathway and its substrate RacGAP1 were activated (22). Activation of RacGap1 lead to the repression of cytoskeleton regulator Rac1, promotion of RhoA activity and cytoskeleton re-organisation to extend pseudopodial protrusions with actin spikes, leading to increased invasion (22). These actin spike extensions depended on activation of the protein FHOD3 by ROCK (23). Part of the role of Rab11-FIP1 in the pseudopodia is dependent on diacylglycerol kinase α (DGK-α) (24). DGK-α phosphorylates diacylglycerol to phosphatidic acid (25). Rab11-FIP1 can interact with phosphatidic acid resulting in the mobilisation of Rab11-FIP1 into the pseudopodia of the cells.

Beside regulating EGFR, we have more recently demonstrated that mutant p53 also promotes the association of Rab11-FIP1 with c-Met (HGFR, hepatocyte growth factor receptor) (26). This interaction promoted the scattering of cells and increased pERK1/2 signalling, contributing to HGF-mediated invasion. To a large extent, all these interactions were dependent on the p53 family member TAp63α and its target gene, the microRNA machinery protein Dicer (27). Combined integrin-RTK signalling is thought to amplify signalling to the Erk1/2 and Akt proteins, driving enhanced invasion, cell scattering and metastasis (16, 17).

As we demonstrated that mutant p53 through Rab11-FIP1 could promote recycling of both EGFR and c-Met, it is tempting to consider that other RTKs are regulated in this manner. Likely candidates could be EphA2, IGF2R, PDGFR and VEGFR. EphA2 has been shown to bind to Rab11-FIP1 to mediate metastasis *in vivo*. In response to HGF, Akt phosphorylates EphA2. In parallel, HGF promotes phosphorylation of Rab11-FIP1 by the Lemur tyrosine kinase-3 (LMTK3) leading to EphA2 binding and plasma membrane expression. This results in cell-cell repulsion, driving metastatic behaviour (10). However, an involvement of mutant p53 was not demonstrated and remains to be elucidated. IGF2R, PDGFR and VEGFR have all been found transcriptionally regulated by GOF mutant p53 (28–31), although a connection with Rab11-FIP1 has not yet been established. Perhaps by both regulating expression as well as actual plasma membrane expression, something that is likely also occurring for β1 integrin (32), mutant p53 could facilitate an amplified cell signalling response that promotes metastastic behaviour and causes the multidrug chemoresistance that is often seen in mutant p53 tumours.

Taken together, these data demonstrate that mutant p53 can regulate integrin/RTK signalling in a Rab11-FIP1-dependent manner to promote invasion and metastasis.

## Mutant p53, Rab11, Rab11-FIP1 and Other Mechanisms Promoting Invasion and Metastasis

Interestingly, mutant p53 was also shown to promote invasion and metastasis by modifying the secretome of cells in a Rab11-FIP1-dependent manner (33, 34). Novo et al. demonstrated that mutant p53 cells with upregulated Rab11-FIP1 are able to influence the phenotype of distant cells through the production of podocalyxin-containing exosomes. These exosomes then remodel the extracellular matrix, supporting invasion of the mutant p53 cells through upregulated Rab11-FIP1 and, notably, α5β1 integrin, c-Met and Transferrin Receptor (TfnR) recycling in neighbouring wildtype p53 or p53-null cells (33).

Additionally, Zhang et al. propose a model in which mutant p53 promotes the vesicular trafficking and secretion of the Hsp90α chaperone in a Rab11-FIP1 dependent manner (35) (**Figure 1**). HSP90α secretion occurs at least in some tumours and cancer cell lines (36–38) and is known to promote tumorigenesis (39–41). The Hsp90α interaction with extracellular matrix proteins and receptors is thought to underlie matrix remodelling and increased invasion and metastasis of mutant p53 cells (35).

In conclusion, these data suggest that Rab11-FIP1 can also act over longer distances through cargo exosome secretion to promote invasion and metastasis.

## Mutant p53 and Rab11-FIP1 Promote Chemoresistance

It has been shown that enhanced integrin signalling confers resistance against several chemotherapeutic compounds (42–44). In cultured lung adenocarcinoma A549 cells, Rab11-FIP1-mediated β1 integrin recycling and signalling was able to confer resistance to cisplatin (45). Various others have shown that increased activation of RTKs *via* integrins confers chemoresistance through RTK signalling (44, 46). As mutant p53 promotes chemoresistance (47–49), it therefore seemed likely that mutant p53 could promote chemoresistance through the Rab11-FIP1/integrin/EGFR signalling pathway. Indeed, cells in which we knocked-out Rab11-FIP1 appeared to become more sensitive to etoposide and cisplatin (18). However, when inhibiting integrins, the sensitivity was less pronounced compared to loss of Rab11-FIP1 expression, suggesting alternative pathways are involved in this chemoresistance (18).

Interestingly, in a screen to detect novel Rab11-FIP1 interacting proteins, we identified the xenobiotic and chemotherapeutic efflux transporter P-glycoprotein (P-gp/MDR1) (18) as well as the copper and cisplatin transporter ATP7B. We could demonstrate that Rab11-FIP1 promoted membrane localisation of P-gp in response to etoposide and cisplatin and enhanced efflux of its substrates (18). Its response to cisplatin is remarkable, as cisplatin is currently not considered a substrate of P-gp. These data suggest a generic response to chemotherapeutics that promotes plasma membrane localisation of Rab11-FIP1 and its cargo (18).

**Figure 2:**
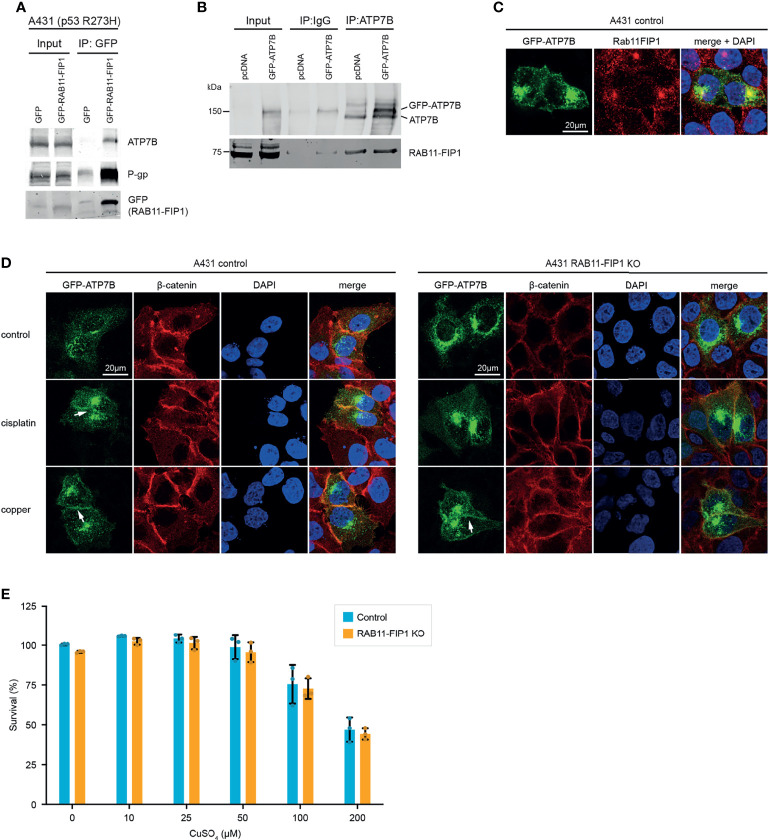
Mutant p53 promotes ATP7B plasma membrane expression in a Rab11-FIP1 dependent manner upon cisplatin stimulation. **(A)** A431 cells expressing mutant p53 (R273H) were transfected to express GFP or GFP-Rab11-FIP1. GFP was immunoprecipitated and co-immunoprecipitation was assessed through western blot, using an ATP7B antibody. **(B)** Co-immunoprecipitation of endogenous ATP7B with Rab11-FIP1 in mutant p53 A431 cells expressing GFP-ATP7B or a GFP control. ATP7B was immunoprecipitated followed by western blot to detect Rab11-FIP1 binding. **(C)** Co-localisation of endogenous Rab11-FIP1 and GFP-ATP7B *(GFP)* was determined using immunofluorescence in A431 cells transfected with GFP-ATP7B **(D)** A431 control or A431 Rab11-FIP1-KO cells transfected with GFP-ATP7B were incubated in cisplatin (3µM) or copper (CuSO_4_, 100µM) for 2 h and assessed for ATP7B localisation. β-catenin was used as membrane marker and DAPI as nuclear marker. Arrows indicate ATP7B plasma membrane expression. All immunofluorescence experiments were performed in triplicates and assessed in >25 cells per experiment and observer with single plane confocal imaging. Representative images are shown. **(E)** A431 control or A431 Rab11-FIP1-KO cells were incubated in increasing copper concentrations for 72 h and subjected to a resazurin survival assay. Error bars indicate standard deviation of 3 independent experiments (n=3). No statistical significance was observed (unpaired t-test) in copper-treated cells. Materials and methods are provided in **Supplemental Data 1**.

ATP7B is a transmembrane protein which translocates from the Golgi apparatus to the plasma membrane in response to copper overload. Through a copper binding domain, ATP7B binds copper and facilitates efflux of excess copper. However, this binding domain is also responsible for the efflux of cisplatin, which could suggest a role for Rab11-FIP1/mutant p53 in promoting cisplatin efflux through this receptor. The Rab11-FIP1/ATP7B interaction was validated in independent immunoprecipitations in A431 cells exogenously (**Figure 2A**) and endogenously (**Figure 2B**) and both proteins colocalise in cells in the Golgi/vesicular compartment (**Figure 2C**). Similar to P-gp, ATP7B accumulated on the plasma membrane of mutant p53 cells in response to cisplatin, but to a lesser extent in Rab11-FIP1 KO cells (**Figure 2D**). These data suggest that in response to cisplatin, Rab11-FIP1 assists the re-localisation of ATP7B to the plasma membrane. Remarkably, loss of Rab11-FIP1 appeared not to limit the amount of ATP7B expressed on the plasma membrane in response to copper (**Figure 2D**) and Rab11-FIP1 KO cells were not more sensitive to copper exposure (**Figure 2E**). These data could indicate that the type of external stimulus (in this case chemotherapeutics, but not copper) dictates Rab11-FIP1 activity.

Interesting from the aspect of chemoresistance is the role of Rab11 in starvation-induced autophagy. Autophagy is known to play a major role in chemoresistance and Rab11 was shown to be required for autophagosome assembly (50). In response to starvation, Rab11 is relocated from recycling endosomes to autophagosomes (51). Mutant p53 is known to inhibit autophagy, but can also itself be targeted for degradation in response to starvation signals (51). It will be interesting to see if the decrease in mutant p53 expression changes the interaction of Rab11-FIP1 with Rab11 and whether this leads to a redistribution of RTKs, integrins or other receptors.

## Other Molecules Regulated by Rab11-FIP1

Rab11-FIP1 has also been shown to interact or co-localise with other proteins and receptors including the TfnR, adiponectin, LRP1B and HIV1 gp160 protein (**Figure 1**), although the functional consequences of these associations remain to be fully elucidated (5, 6, 52–54). Of these, the Rab11-FIP1-dependent regulation of the TfnR, mostly involved in iron uptake, has been most thoroughly studied, but whether Rab11-FIP1 has a role in iron homeostasis is unknown. Interestingly, mutant p53 cells are likely to have elevated iron levels and mutant p53 expression is correlated to elevated transferrin expression (55).

These data could point to a role for Rab11-FIP1 in mediating several distinct downstream signalling pathways downstream of mutant p53 and would make Rab11-FIP1 an interesting target for therapeutic intervention and raises the question of how Rab11-FIP1 is regulated within the cell.

## Intracellular Regulation of Rab11-FIP1

In many of the previous settings, it appears that Rab11-FIP1 function can be altered dependent on the stimulus impacted upon cells. In response to EGF, Rab11-FIP1 regulates EGFR membrane expression and in response to etoposide or cisplatin, Rab11-FIP1 promotes expression of P-gp and/or ATP7B to the plasma membrane. Interestingly, Francavilla et al. demonstrated that EGFR recycling can be dependent on the type of signalling (20). When stimulated with TGF-α, EGFR recycling occurred in a Rab11-FIP1 -dependent manner. However, upon EGF incubation, EGFR was recycled in a Rab7-dependent manner, itself dependent on phosphorylation.

The activity of Rab11-FIP1 has been shown regulated by phosphorylation through 2 different kinases so far: Lemur Tyrosine Kinase 3 (LMTK3) and MAP/Microtubule Affinity-Regulating Kinase 2 (MARK2).The LMTK3 kinase promotes S435 phosphorylation of Rab11-FIP1 in response to HGF stimulation (10) and has been studied for its role in breast cancer (56).The MARK2 kinase was shown to promote phosphorylation of S234 in Rab11-FIP1 and promoted polarization of MDCK cells upon a calcium switch. MARK2 is overexpressed in cisplatin-resistant cell lines and expression level of MARK2 correlate with resistance to cisplatin in non-small cell lung cancer (57). This finding suggests that the increase in Rab11-FIP1 activity in mutant p53 cells may be mediated by the MARK2 kinases.

While the activity or expression of these kinases has not directly been linked to mutant p53 expression, it is easy to hypothesise that an increase of Rab11-FIP1 activity could be facilitated through mutant p53-dependent activation of the aforementioned kinases. Several other kinases such as MAP2K3 (58), Aurora kinase A (59) and JNK (60) are regulated by or cooperate with mutant p53. Enhancing the activity of kinases that phosphorylate Rab11-FIP1 would lead to an increased Rab11-FIP1 activity, favouring invasive growth and chemoresistance.

## Targeting the Rab11-FIP1 Signalling Pathway for Cancer Treatment

With Rab11-FIP1 constituting what appears to be a distribution hub enabling pro-tumorigenic effects through different processes, it could be an interesting drug target in a mutant p53 setting, especially given the fact that mutant p53 targeting therapies are not yet available in the clinic. Several avenues could be explored and can be divided into therapies targeting Rab11-FIP1 itself or any of its downstream effector molecules.

In 2005, Marie et al. reported that Rab11-FIP1 is degraded by calpains in a calcium-dependent manner (61). Increased intracellular Calcium levels (by using the ionophore Ionomycin) reduced Rab11-FIP1 levels. Ionophores such as Ionomycin have been explored as potential anticancer drugs, and might contribute to apoptosis due to increased calcium levels in synergy with chemotherapeutics (62). It will be interesting to explore this strategy in cancers that depend on Rab11-FIP1 and/or mutant p53 expression.

Another way in which Rab11-FIP1 expression levels can be regulated is by the microRNA miR-93. Rab11-FIP1 is a direct target, as demonstrated in cervical cancers in which elevated miR-93 levels coincide with reduced Rab11-FIP1 levels (11). Using cultured cells, Zhang et al. showed that the knockdown of miR-93, allowed for higher Rab11-FIP1 expression, increases apoptosis and reduces proliferation. In that context, miR-93 knockdown seems an interesting approach to reduce tumorigenicity by acting on Rab11-FIP1. However, these findings go against the current “dogma” in the field where elevated Rab11-FIP1 levels and its activation are tumorigenic (11) and might therefore indicate a tissue specific effect, making it pivotal to study Rab11-FIP1’s role in different cancers. In other cancers, using miR-93-containing constructs as actual therapeutic could be a strategy now that siRNA therapy has FDA approval to be used in the clinic (63).

Downstream of Rab11-FIP1, EGFR and/or integrin inhibitors have been investigated. Resveratrol and curcumin, which are known to reduce tumour growth, impacted on the expression or activation of these proteins. In oral squamous cell carcinoma development and invasion, Rab11-FIP1 upregulated Zeb1, and subsequently MT1-MMP downstream of β1-integrin/EGFR and β-catenin signalling. Resveratrol inhibited EGFR activation and β1 integrin recycling (21). In cultured SKOV-3 and PA-1 ovarian cancer cells, Rab11-FIP1 promoted invasion by stabilising β1 integrin and activating FAK through EGFR. Interestingly, Curcumin reduced β1 integrin stability, thus reducing EGFR and FAK activation, leading to reduced invasion (64). Most interestingly, resveratrol and curcumin have also been demonstrated to inhibit tumorigenicity of mutant p53 expressing cells. The Silva group has shown in cultured breast cancer cells that Resveratrol inhibits mutant p53 aggregation, but also cell proliferation and migration, thereby reducing tumorigenicity (65). Curcumin was able to re-activate mutant p53 to induce cell death in cultured cells (66, 67), as well as reduce growth of tumour xenografts (66).

Other inhibitors to consider in this respect are direct EGFR inhibitors such as Gefitinib, Erlotinib, Panitumumab and Cetuximab or the integrin inhibitor Cilengitide, which have all been in clinical trials, with some more successful than others. Perhaps, those work best in a setting of mutant p53 and Rab11-FIP1 in which cancer cells are dependent or even addicted to the amplified signalling.

## Conclusion and Future Perspectives

In this perspective, we have seen that mutant p53 can regulate Rab11-FIP1 to modulate a plethora of proteins involved in tumour formation, invasion, metastasis and chemoresistance. Any molecule that could inhibit Rab11-FIP1 could therefore have the potential to stop tumour growth, prevent metastasis and prevent chemoresistance. Some molecules that inhibit downstream pathways of Rab11-FIP1 have shown potential, but strategies that would act on Rab11-FIP1 itself would presumably be more potent. Of interest are the regulation of Rab11-FIP1 by calpains and the degradation upon ionomycin treatment, as well as the potential of targeting Rab11-FIP1 by siRNA. In order to develop a Rab11-FIP1 -based therapy, more research will be needed into the regulation of Rab11-FIP1, its interaction with cargo in different conditions and the cancer-specificity of this response.

## Data Availability Statement

The original contributions presented in the study are included in the article/**Supplementary Material**, further inquiries can be directed to the corresponding author.

## Author Contributions

YvG, conceptualization, visualisation, and writing – original draft. VP and LA, conceptualization and investigation. PM, conceptualization, funding acquisition, methodology, project administration, visualisation, and writing – original draft. All authors contributed to the article and approved the submitted version.

## Funding

PM and VP were funded through an HDF fellowship (WT101242AIA) of the Wellcome Trust. LA was supported by the MRC Toxicology Unit. PM and YvG were supported by CRUK core funding (C5759/A27412). PM is currently supported by Durham University.

## Conflict of Interest

Author VP was employed by company Avacta Life Sciences. Author LA was employed by company Orbit Discovery.

The remaining authors declare that the research was conducted in the absence of any commercial or financial relationships that could be construed as a potential conflict of interest.

## Publisher’s Note

All claims expressed in this article are solely those of the authors and do not necessarily represent those of their affiliated organizations, or those of the publisher, the editors and the reviewers. Any product that may be evaluated in this article, or claim that may be made by its manufacturer, is not guaranteed or endorsed by the publisher.

## Abbreviations

Akt, Protein Kinase B; ATP7B, Copper-transporting ATPase 2; DGK- α, Diacylglycerol Kinase alpha; EGF/EGFR, Epidermal Growth Factor (Receptor); FAK, Focal Adhesion Kinase 1; FHOD3, Formin Homology 2 Domain Containing 3; HGF/HGFR, Hepatocyte Growth Factor (Receptor); IGF2R, Insulin Like Growth Factor 2 Receptor; LMTK3, Lemur Tyrosine Kinase 3; MARK2, Microtubule Affinity Regulating Kinase 2; MT1-MMP, Membrane-Type 1 Matrix Metalloproteinase; P-gp, P-glycoprotein; PDGFR, Platelet-Derived Growth Factor Receptor; Rab11-FIP1, Rab11 Family-Interacting Protein 1; RCP, Rab Coupling Protein; ROCK, Rho-associated Protein Kinase; RTK, Receptor Tyrosine Kinase; TfnR, Transferrin Receptor; VEGFR, Vascular Endothelial Growth Factor Receptor.
