# Supplementary material for: Rab11-FIP1/RCP Functions as a Major Signalling Hub in the Oncogenic Roles of Mutant p53 in Cancer
Source: Front Oncol. 2021 Dec 21;11:804107. doi: 10.3389/fonc.2021.804107 (PMC9231559; doi:10.3389/fonc.2021.804107)
Supplement: Supplementary file 1 [file DataSheet_1.docx]

**Materials and Methods**

**Cell lines and culture conditions**

A431 epidermal carcinoma cells (expressing mutant p53 R273H) were sourced from ATCC (www.ATCC.org) and were cultured in DMEM (Gibco 41966) supplemented with 10% FBS (Sigma F7524) and 5% penicillin/streptomycin (Thermo Fisher Scientific) in 5% CO_2_ atmosphere at 37°C.

Generation of A431 Rab11FIP1-KO cells was described before^1^

In treated conditions, cells were incubated with cisplatin (3 μM) or CuSO_4_ (10-200 μM) for 2 h or 72h for cell survival assays.

**Transfection**

A431 cells were reverse transfected with 2 μg GFP or 5 μg GFP-ATP7B using 10 μl Lipofectamine 2000 (Invitrogen) in 6 well plates by mixing DNA and lipofectamine directly in serum free medium, vortexing and incubating at RT (15 min) before adding dropwise to cells.

**Immunoprecipitation**

Immunoprecipitation of GFP was described before^2^. Immunoprecipitation of ATP7B was achieved by lysing cells from a 60 cm^2^ dish in 200 µL of non-denaturing lysis buffer (NDLB: 5 M NaCl, 1 M Tris-HCl, pH 7.5, 0.5 M EDTA, 0.5 M EGTA, 0.5 M NaF, 0.1 M NaVO4) with complete protease inhibitor (Sigma), 0.15% Tween 20 (Santa Cruz) and 10% SIGMAFAST Protease Inhibitor Cocktail Tablet (Sigma). Scraped cells were then forced 5 times through a 27.5 G needle. Supernatant was collected after centrifugation (17.000xg, 15 min, 4 °C). For each sample, 30 µL of Protein G Dynabeads (Thermo Scientific) were coupled with antibodies raised against GFP (0.75 µg, Abcam ab6556 ), IgG (1 µg, Santa Cruz Biotechnology) or ATP7B (1 µg, GeneTex GTX30639) for 1 hr at 4 °C. Antibody-coupled beads were then incubated with the protein lysates with agitation (16h, 4 °C). Unbound lysate was washed away 4X with NDLB, immunoprecipitate was eluted in 30 µL of 2x reducing sample buffer and boiled for 5 min.

**Western blot analysis**

Western blot was described previously^3^. Lysates were run on SDS-PAGE and blotted on nitrocellulose. The blots were probed with the following primary antibodies: ATP7B (GeneTex GTX30639), Rab11-FIP1 (Sigma-Aldrich GW21574A), P-gp (G-1, Santa Cruz Biotechnology [sc-13131](https://www.scbt.com/p/mdr1-antibody-g-1?requestFrom=search)). Secondary antibodies for ATP7B and Rab11-FIP1 were donkey-anti-mouse IRDye 800CW (Li-Cor Biosciences), imaged using the Li-Cor Odyssey SA platform. Secondary antibodies for P-gp were anti-mouse HRP-conjugated (Santa Cruz Biotechnology). Blots for P-gp expression were developed with SuperSignal Western Blot Enhancer (Thermo Scientific).

**Immunofluorescence and confocal imaging**

Immunofluorescence was described previously^3^. Cells were grown on glass cover slips in 24 well cell culture plates until reaching 70% confluency, washed with ice-cold PBS and fixed with 4% paraformaldehyde (Thermo scientific) at 4 °C (15 min). Cells were washed with PBS and permeabilised with 0.1% Triton-X at RT (10 min), blocked in 5% BSA (Sigma) at RT (1 h), followed by incubation with primary antibody in block buffer at 4 °C (overnight), and secondary antibody (1 h). Following primary antibodies were used: GFP (Abcam), Rab11-FIP1 (Cell Signalling D9D8P), β-catenin (abcam ab16051). All immunofluorescence experiments were performed in triplicates and assessed in >10 fields per experiment and observed with single plane confocal imaging (Zeiss LSM800).

**Cell viability tests**

Cell viability was determined by using resazurin on a Molecular Devices Spectramax M5 at 575 nm fluorescence (Excitation at 555 nm). 5000 cells per well were seeded (48 h after siRNA transfection) in 96-well plates and incubated with CuSO_4_ (72 h). Measurements were done as three technical replicates in three independent experiments.

References

1. Mackay, H.L. *et al.* Genomic instability in mutant p53 cancer cells upon entotic engulfment. *Nature communications* **9**, 3070 (2018).

2. Muller, P.A. *et al.* Mutant p53 drives invasion by promoting integrin recycling. *Cell* **139**, 1327-1341 (2009).

3. Muller, P.A. *et al.* Mutant p53 enhances MET trafficking and signalling to drive cell scattering and invasion. *Oncogene* **doi:10.1038** (2012).
